# Supplementary material for: Prediction of Cancer Proneness under Influence of X-rays with Four DNA Mutability and/or Three Cellular Proliferation Assays
Source: Cancers (Basel). 2024 Sep 18;16(18):3188. doi: 10.3390/cancers16183188 (PMC11430126; doi:10.3390/cancers16183188)
Supplement: Supplementary file 1 [file cancers-16-03188-s001.zip › cancers-3171888-supplementary.pdf]

Prediction of Cancer Proneness under Influence of X-rays with Four DNA Mutability and/or Three Cellular Proliferation Assays

Laura El Nachef [1], Larry Bodgi [1,2,3], Maxime Estavoyer [4], Simon Buré [4], Anne-Catherine Jallas [1], Adeline Granzotto [1], Juliette Restier-Verlet [1], Laurène Sonzogni [1], Joëlle Al-Choboq [1], Michel Bourguignon [1,5], Laurent Pujo-Menjouet [4] and Nicolas Foray [1,*]

Supplementary data

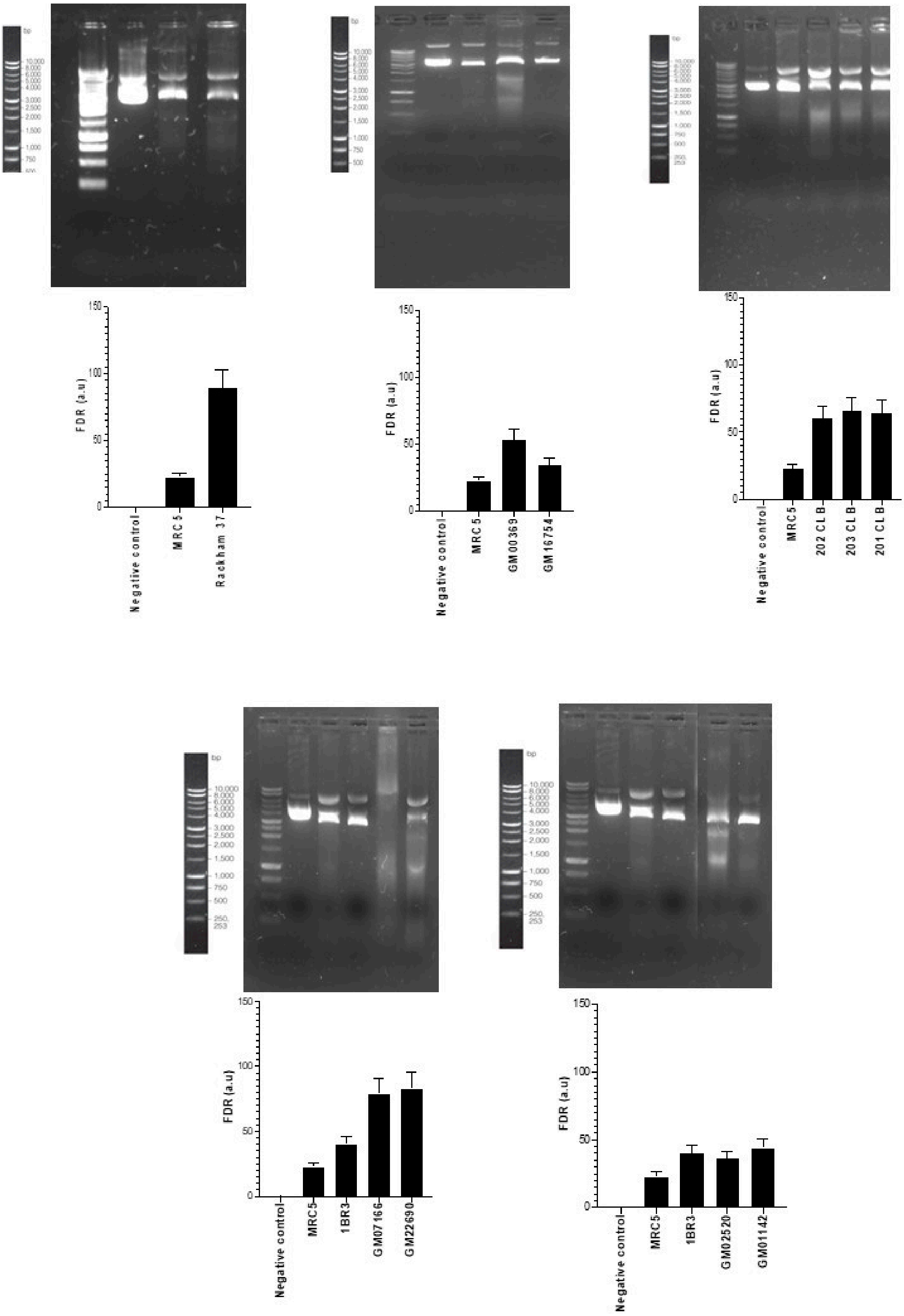

Figure S1 : Representative images of electrophoresis gels after nuclease plasmid assay with the indicated cell lines. The fraction of DNA released (FDR) has been quantified in arbitrary units (a.u).

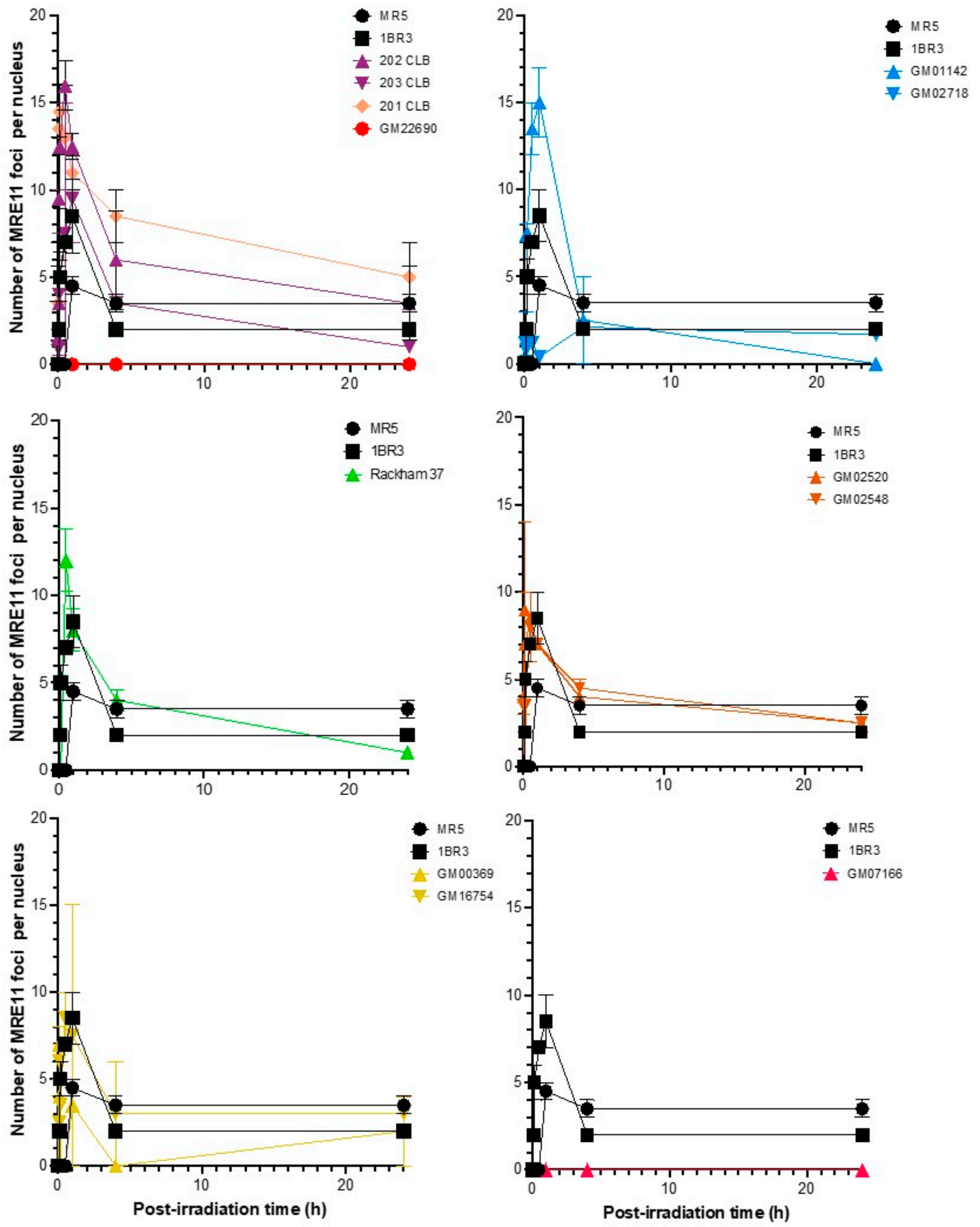

Figure S2 : Number of MRE11 foci per nucleus plotted against the post-irradiation time (2 Gy X-rays). Each plot corresponding to the indicated cell line represents the mean ± standard error of the mean (SEM) of three independent replicates, at least.

Table S1 : Numerical values for all the endpoints used in this study

Syndromes	ERR	Plasmid assay	HPRT assay	Nuclease assay	MRE11 assay	%G2/M	%G1	Escape (%)	HxG
Control	0	3.2	10	22.7	5	1.1	0	0	
BRCA1	0.4	27	26	60	14	1	0	0.63	
BRCA2	1	25	-	65	12	1	3	1.5	
RB	3.2	32	57	44	7.4	3	4	15.9	
BLM	2.4	31.5	34		8.25	4	0	18.2	
FANC	1.4	35	36	53	8	1.4	0	17	
NF1	3.6	42	60	-	9	4	0	17	
NBS	7	46	-	55	0	9	1.6	14	
AT	9	56	68	63	0	10	19	50	

Table S2 : Origin of the ERR values used in this study*

Syndromes	ERR	References
Apparently healthy	0	-
BRCA1	0.4	Average of [33] [34] [35]
BRCA2	1	[34]
RB	3.2	[36]
BLM	2.4	[37]
FANC	1.4	[38]
NF1	3.6	[39]
NBS	7	[40]
AT	9	[41]

*by considering that ERR = RR-1
